# Fire Risk Assessments of Informal Settlements Based on Fire Risk Index and Bayesian Network

**DOI:** 10.3390/ijerph192315689

**Published:** 2022-11-25

**Authors:** Jun Hu, Xuecai Xie, Xueming Shu, Shifei Shen, Xiaoyong Ni, Lei Zhang

**Affiliations:** 1 School of National Safety and Emergency Management, Beijing Normal University at Zhuhai, Zhuhai 519087, China; 2 Academy of Disaster Reduction and Emergency Management, Ministry of Emergency Management & Ministry of Education, Beijing Normal University, Beijing 100875, China; 3 Department of Engineering Physics, Institute of Public Safety Research, Tsinghua University, Beijing 100084, China; 4 Beijing Key Laboratory of City Integrated Emergency Response Science, Tsinghua University, Beijing 100084, China

**Keywords:** informal settlements, fire risk, risk index, Bayesian network

## Abstract

The specific risk assessment of informal settlements (IS) is important for the management of IS and protection of environmental safety and public health. In this paper, we introduced the different types of IS in China, and conducted the fire risk assessment on 26 burning buildings in these IS, providing a semi-quantitative and scenario fire risk perception of IS in China for the readers. Two methods, the risk index and the Bayesian network, are proposed and adopted for the fire risk assessment in IS. First, a risk index system with a total of 69 factors is used to assess the degree of fire risk of buildings in IS semi-quantitatively, and the result shows that fire equipment and fire safety management on IS are seriously lacking. Then, a Bayesian network of building fire risk with a total of 66 nodes was established to assess the fire risk from ignition to spread as well as the safety evacuation. Overall, the possibility of ignition is high, but due to the role of fire equipment and fire protection design, the possibilities of fire from ignition to spread is gradually reduced. Finally, we also put forward some feasible suggestions for occupants in IS, community organizations and emergency managers to reduce the fire risk from the aspects of fire equipment and fire safety management.

## 1. Introduction

In many low- and middle-income countries, there are some informal settlements (IS) in cities. Broadly speaking, informal settlements are places built outside land-use schemes and without planning permission [1]. In some literature, the “informal settlements” mainly refer to slums or shantytowns in some African countries or Asian countries, and informal settlements, slums, squatter settlements, unplanned towns and shantytowns are terms that are used interchangeably in the literature. From the perspective of risk, the fire risk in IS is at a high level, and it is of great significance to conduct fire risk assessments and understand the risk sources in IS.

Nowadays, many studies on IS fires have been carried out, including fire dynamics [2,3,4], human behaviors [5,6], fire detection [7] and fire investigation [8]. When it comes to fire risk, David Rush et al. have examined fire risks in IS in New Delhi, Cape Town and Lebanon, based on literature, statistical data and qualitative interviews [9]; Natalia Flores Quiroz et al. have obtained fire risk perception of the IS complex inhabitants with surveys to comprehend why the fire started [10]; Richard et al. have qualitatively presented an appraisal of various interventions and strategies to improve fire safety in IS in South Africa [11]; Isabela et al. have assessed the fire exposure and risks in IS in Tanzania, mainly based on interviews [12]; Morrissey and Taylor have analyzed the factors influencing the fire risk in the IS in Cape Town qualitatively [13]. In addition to fires, considering other disasters, such as flooding or earthquake, some studies have also analyzed the comprehensive risks in IS [14,15,16,17].

Overall, the discussion and analysis of the fire risk of IS are qualitative in these studies. The fire risks in IS have been discussed to be high, but the degree of “high” has not been quantified. Some scholars have also put forward some ideas of quantitative or semi-quantitative risk assessment; for example, John Twigg et al. have suggested that community-based risk and vulnerability assessment methods could be adapted, Matthew et al. have developed a theoretical framework coupling “disaster hazards”, “vulnerability” and “informal settlements” to conceptualize a detailed risk profile in IS, and Giambelli et al. have presented an idea for the practical decision support system for the IS in Kathmandu [1,18,19]. However, the specific assessment has yet to be carried out, which is partly due to the lack of relevant information of IS in these countries [18].

Meanwhile, in addition to the slums, squatter settlements and shantytowns, other IS types should also be studied. IS are not going to disappear completely within a short period in developing countries; instead, the form of IS is constantly changing. In China, with the development of the economy, the number of slums, squatter settlements or shantytowns has disappeared, but there are still some settlements built outside land-use schemes or without planning permission, and they are composed mainly of buildings that deviate from the standard building regulations. Compared to the IS in other countries, the IS in China have some different features, and the types of IS are likely to emerge in other developing countries in the future as society develops. Hence, the research of fire risk in IS in China is meaningful and prospective, as it can expand the scope of current research on IS and provide experience on fire prevention for IS in other developing countries.

In brief, the research on fire risk in informal settlements is still insufficient. On one hand, the categories of IS can be expanded more than slums, squatter settlements and shantytowns; on the other hand, the fire risk can be assessed in more depth. Recently, the Bayesian network (BN) has been applied in the field of fire risk assessment to reflect the uncertain characteristic of risk [20,21,22,23]. Therefore, the fire risk of IS can be further quantified and assessed, so as to perceive the specific degree of risk and how the fire risk changes in different stages.

Accordingly, we carry out a semi-quantitative and staged fire risk assessment for IS in China with two methods, namely the risk index and the Bayesian network. The risk index is based on an index system currently widely used in China’s fire protection market, and it can clarify the sources of risks in IS and characterize the degree of high risk; while with the Bayesian network, the fire risk can be analyzed from ignition to spread. This research is helpful to supplement the current research on fire risk in IS and provide valuable experience on fire prevention for IS in other developing countries. The paper is organized as follows: Section 2 introduces the types and characteristics of IS in China and provides brief information on selected IS fire cases. Section 3 introduces the two risk assessment methods, namely the risk index and the Bayesian network. Section 4 provides the risk assessment results of IS in China with the two methods mentioned above, and Section 5 makes a detailed discussion of the fire risk and puts some feasible suggestions to reduce the risk. Finally, Section 6 concludes this paper.

## 2. IS Types and Fire Cases

### 2.1. IS Types in China

**Figure 1:**
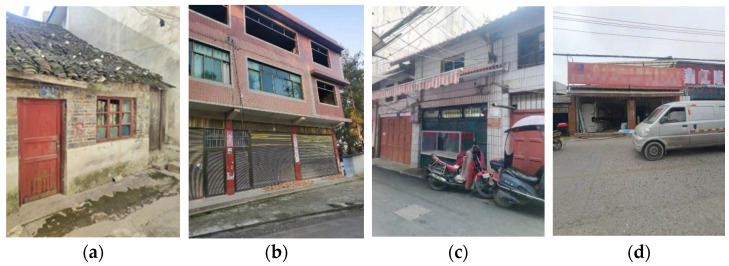
Four types of IS in China. (**a**) Old communities, where the main body of the building was made of bricks. (**b**) Informally constructed settlements, where the buildings were constructed without official approval. (**c**) Informally modified settlements, where the additional decoration of the roof was private and illegal. (**d**) Informally functioned settlements, where the functions of accommodation, production, storage and business were mixed.

By the end of 2018, more than 100 million shantytown residents had left the shantytowns and lived in the planned buildings in China [24]. However, there are still some other types of IS in China, which are composed mainly of buildings that deviate from the standard building regulations. Considering the risk of fire, the IS in China are categorized as follows, and some typical constructions are shown in Figure 1:

1. Old communities (OC): The formation of these communities is legal and planned, but the construction of the buildings is relatively old. The old communities have a great fire hazard, as the construction materials (such as wood and brick) and fire protection design (such as fire separation distance) meet the previous standards, but do not comply with the current fire regulations.
2. Informally constructed settlements (ICS): These settlements mostly appear in urban–rural fringe areas or villages in the city (VIC, a special phenomenon of urbanization in China), and some buildings were constructed without official approval. After construction, they have not been checked or audited by the fire department, so they are fragile in the event of a fire.
3. Informally modified settlements (IMS): In these settlements, the construction of the buildings was approved, but the later modifications, such as additional floors, decoration processes or annexes, were private and illegal. After the modifications, the fire hazards of the building increased. For example, some buildings used cheap but flammable color steel plates as roofs.
4. Informally functioned settlements (IFS): In these settlements, the construction of the buildings was approved, but the owners changed the function of the buildings without authorization, which greatly increased the fire risk. For example, in some residential buildings, the functions of accommodation, production, storage and business are mixed, leading to the increase in flammable substances. These settlements are also called “mixed-function settlements” in China, and they violate the regulations of fire safety requirement for places combining habitation, production, storage and business (GA703-2007) [25].

### 2.2. Fire Cases

To carry out fire risk assessment on IS, it is necessary to obtain comprehensive information of the objects. However, it is hard to obtain the comprehensive information in IS [17]. In this research, we selected 26 building fire cases in IS in China for risk assessment. For one thing, the detailed information of these serious fire cases can be found in the investigation report issued by the fire department [26], based on which the specific risk assessment can be conducted. For another, the result of burning is also a concentrated reflection of the high-risk characteristics of IS. The brief information of the fire cases is shown in Table 1.

These fire cases occurred in the above-mentioned four types of IS in different areas of China (to the city level) from 2011 to 2018. In these cases, the causes of the fire include electrical failure, arson and careless use of fire, and many fires have caused serious casualties. In particular, the common cause of some electrical fires in IS is a short circuit caused by electric bicycle charging (e.g., Case 3, 4, 19 and 22). In fact, not only for IS, but the fires also caused by electric bicycles account for a large proportion of the current number of fires in China. The electric bicycle has served as a means of transport for many families, as it is cheap and labor-saving. However, many fires are caused by the excessive charging time or the unqualified battery [27].

## 3. Risk Assessment Methods

### 3.1. Fire Risk Index

Fire risk index is a widely applied method in the field of fire risk assessment. With this method, the value called “risk index” is a measure of the level of safety/risk in the evaluation object. First, the fire risk index system is composed of many factors which are selected by experts, including scientists, insurance, fire brigade, etc. Second, the importance of each factor is decided by assigning a value. This value is based on the knowledge and the experience of experts with a weight assignment method such as the analytic hierarchy process (AHP) and fuzzy mathematical model [28,29]. Meanwhile, the state of each factor is determined with another value, representing the grade of each factor. Finally, these values are operated by some combination of arithmetic functions to achieve a single value, and that is risk index. A popular arithmetic function is shown in Formula (1):

(1) $$I=\overset{n}{\underset{i=1}{\sum }}w_{i}x_{i}$$

where I denotes the risk index, *n* denotes the total number of factors, $w_{i}$ is the weight of factor *i,* and $x_{i}$ is the grade for factor *i*.

The fire risk index system, including the factors and weights, varies depending on the country/region and building type. Some studies have built risk index systems for the fire risk in IS [30,31,32,33], but the risk index systems are quite different for different IS cases, and the risk characteristics of IS are not summarized under the same evaluation system. In this research, we chose a fire risk index system developed by Global Safety Tanzer Technology Company. This fire risk index system is widely applicable for different buildings, as it has been used for fire risk assessment on more than 50,000 households and businesses in China and affirmed and recognized by the users [34]. With this index system, the risk characteristics of IS can be summarized under the same evaluation system. This system is composed of 69 factors, which are selected by the experts based on the fire regulations in China and considering the availability of information, and the weight of each factor is decided by experts with the AHP method. The factors are structured under three global factors: fire protection design, fire equipment and fire safety management. The total sub-factors, weights and assignment rules are specifically shown in Appendix A. Meanwhile, it should be noted that the “fire risk index” is a measure of the level of safety in this paper, that is, the higher the index value, the lower the risk. In this way, the safest state corresponds to a score of 100 points, and the most unsafe state corresponds to 0. Both 100 points and 0 points are idealized states and do not exist in practice. The score ratio of each global factor is shown in Table 2. In this paper, the risk index and safety score are the same thing.

### 3.2. Bayesian Network

The ending scene of a building fire is uncertain. It may be a small fire extinguished at the early stage after ignition, or a big fire extinguished after full development or even spread to the neighboring buildings. During the whole process of burning, the measures including fire prevention and firefighting will be involved, making the possibility of small fire to big fire continuously reduced. Meanwhile, the reliability of fire prevention and firefighting (fire doors, sprinkler systems, etc.) is uncertain, as they are affected by many factors. Therefore, the fire risk changes in stages.

With the fire risk index, the risk sources of fire and their impacts can be understood comprehensively, but it cannot reflect the stage characteristics of fire risk. Bayesian network (BN) is a tool for uncertainty reasoning based on graph theory, and it consists of a directed acyclic graph (DAG) and conditional probability tables (CPT). The DAG displays the relationship between various variables qualitatively, while the CPT express their relationship quantitatively. BN has been widely used in the field of risk assessment [20,21,22,23], and in some research, it is used to assess the risk at different fire stages [22,23,35].

**Figure 2:**
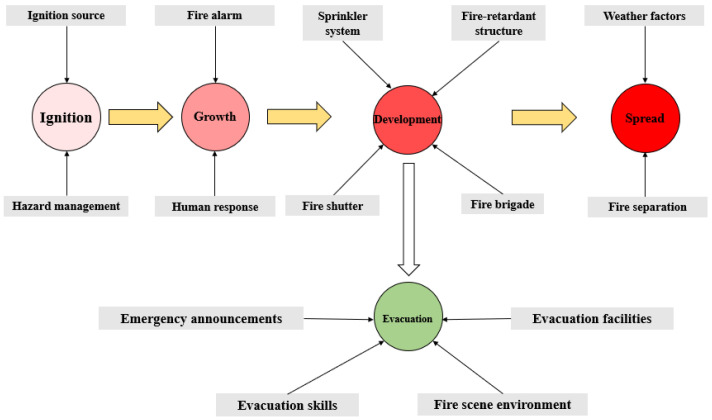
Conceptual framework for fire risk assessment based on BN.

In this study, the conceptual framework for fire risk assessment based on the Bayesian network is shown in Figure 2. According to the intensity of combustion, a fire is divided into the following major stages: ignition, growth, development and spread. The “growth” stage represents the early stage after the ignition, when the fire is small and can be extinguished by personnel; the “development” stage indicates that the fire is bigger and difficult to be controlled by personnel (building occupants, staff, but not including professional firefighters), and it burns fully inside the building; the “spread” stage means the fire is so big that it can spread to the neighboring buildings. In this way, the fire risk is characterized by the possibilities of the fire in different stages, as well as the possibility of safety evacuation under different situations (the prevention of casualties in building fires is more important, so we only consider evacuation, while other consequences such as property damage and building collapse are not considered in this paper). Generally, the possibility of ignition is related to the ignition sources and hazard management; the possibility of growth is related to the reliability of fire alarm systems and human response after ignition; the possibility of development is related to the reliability of fire control measures such as fire shutter, sprinkler system, fire-retardant structure and response of the fire brigade; the possibility of spread is related to the fire separation and weather factors (mainly wind speed); and the possibility of safety evacuation is related to emergency announcements, evacuation facilities, evacuation skills and the fire scene environment when the fire is out of human control.

**Figure 3:**
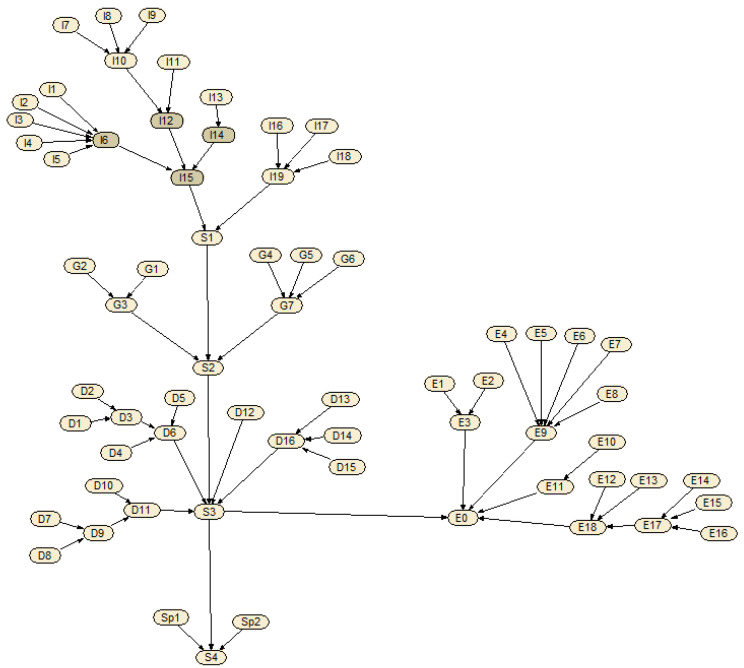
The Bayesian network structure for fire risk assessment with the software of Netica.

Furthermore, these related factors are affected by sub-factors. For example, whether the fire source exists is related to whether the residents are smoking, using open flames and so on. In total, there are 66 nodes in the Bayesian network for fire risk assessment, and the Bayesian network structure constructed with the software of Netica is shown in Figure 3. The detailed names and states of these nodes are shown in Appendix B.

After the construction of Bayesian network structure, it is necessary to set the structural parameters, namely the conditional probabilities, which represent the quantitative relationship between nodes. For the Bayesian network established in this study, the parameters are set based on the knowledge and experience of experts. The correspondence between the orientation of experts and the quantitative possibility value is shown in Table 3. Although the treatment seems subjective, it is widely used in the current research as the conditional probability is difficult to objectively determine [21,22,35].

## 4. Results

According to the investigation report issued by the fire department, we can obtain the comprehensive information of the burning cases in IS. Then the grades for factors in the fire risk index system and the states of nodes in the Bayesian network can be obtained. Risk assessment can be carried out by using fire risk index and BN methods.

### 4.1. Fire Risk Index in IS

**Figure 4:**
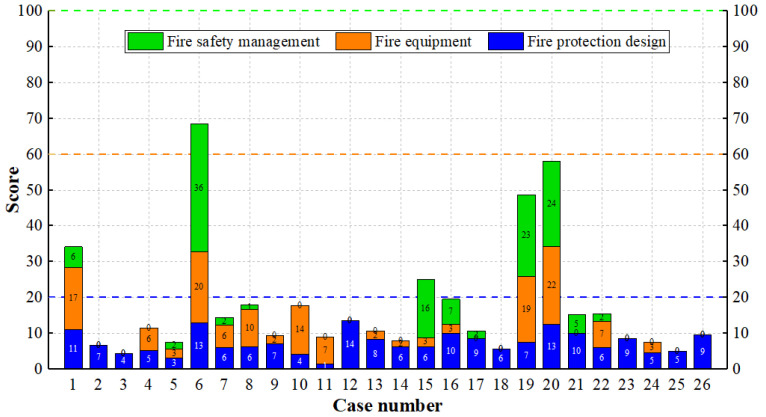
Safety scores of burning cases in IS. Fire equipment and fire safety management are seriously lacking in some cases.

Based on the grades and weights of factors in the fire risk index system, the fire risk indexes of the 26 burning cases in IS can be obtained, and the results are shown in Figure 4. Generally speaking, a score below 60 means that the building is unsafe. Obviously, the risk of these fire cases is high, and the safety score is low. Except for Case 6, which has a significantly higher score, the safety scores of the other 25 cases did not exceed 60 points (The cause of Case 6 is arson, and the building was equipped with some fire facilities, although the construction of the building is illegal).

It can be seen that the scores for fire protection design of many cases are no more than 10 points. This is related to the building materials with low fire resistance ratings and inadequate design of fire separation and evacuation. The fire equipment and fire safety management are seriously lacking for some cases, as one of them has a score of 0 (Case 4, 9 and 10), or even both are 0 (Case 2, 3, 25 and 26). The complete fire equipment includes a water supply system, fire hydrant system, sprinkler system, automatic fire alarm system, smoke management system, emergency lighting system, evacuation indicator system and fire extinguishers. But for many buildings in IS, there was no fire equipment, or the fire equipment was incomplete, and thus the safety score for this item is low. Fire safety management has not received enough attention in IS, leading to its low safety score. The fire control publicity and training provided by the communities are inadequate in IS: on one hand, people have little knowledge of requirements on safe ignition (Case 4, 11, 21 and 26). On the other hand, there is no training and drill for extinguishment or evacuation, resulting in the improper response to the fire at an early stage (Case 21 and 25) and incorrect escape ways when the fire becomes big (Case 4, 11 and 17).

### 4.2. Fire Risk in Stages with BN

**Figure 5:**
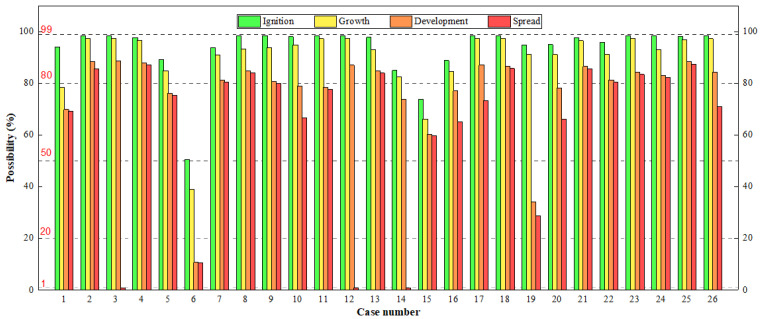
Possibilities from ignition to spread of burning cases in IS. It is gradually reduced.

**Figure 6:**
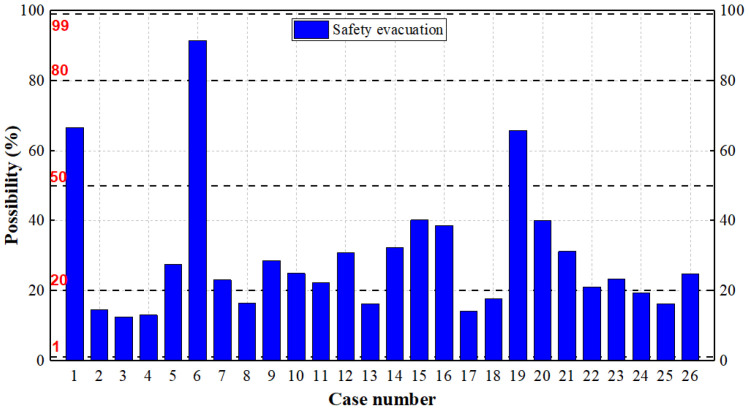
Possibilities for safety evacuation of burning cases in IS. The possibility of Case 6 is the highest.

According to the Bayesian network, the possibilities from ignition to spread, and the safety evacuation are analyzed. Overall, the probability value of 20% or less can be considered as safe, according to the criteria in Table 3. The results are shown in Figure 5 and Figure 6. It can be seen that the possibility of ignition in IS is high, but due to the role of fire equipment and fire protection design, the possibilities of a fire from ignition to spread is gradually reduced. If the fire protection design is better and the reliability of the fire equipment is higher, the reduction process is more obvious (Case 6 and 19). Moreover, the results are consistent with the results of the fire risk index method. The safety score of Case 6 is the highest among all the cases with the fire risk index, while the possibility from ignition to spread is the lowest, and the possibility of safety evacuation is the highest for Case 6 with the Bayesian network. The cases with lower safety scores in the fire risk index, such as Cases 2, 3, 25 and 26, have higher possibilities from ignition to spread, and the possibilities of safety evacuation is also lower, correspondingly, with the Bayesian network.

The high fire risk of IS is specifically explained from the fire process and evacuation:

1. Ignition: Judging from the causes of IS fires in Table 1, 18 of these cases were electrical fires. The electrical wiring in IS is mostly irregular, and, coupled with improper operation or no separation of combustible materials (Case 9, 10 and 24), the possibility of fire is high.
2. Fire growth: After the ignition, a fire can be extinguished in the stage of growth. Two conditions need to be satisfied for extinguishing: the fire is discovered in time, and the human response is correct. However, in the fire cases, the two conditions were not met. Some fires occurred during the night when people were asleep, and there is no fire detector installed, so the fire is hardly detected in time. Secondly, the fire extinguishers were extremely lacking in IS, and the residents did not receive training on how to extinguish a fire. Therefore, the extinguishment is unlikely to succeed, and the possibility of growth is high.
3. Fire development: If there is a fire compartment or sprinkler system in the building, or the fire brigade can arrive timely, the possibility of fire development can be reduced significantly. For many burning cases in IS, these is no fire compartment or sprinkler system (or it is damaged), and thus the prevention of the fire development mainly depends on the fire brigade. However, many IS are far away from the fire brigades, so it took a long time for the fire brigade to reach the fire site. In only four of the 26 fires, firefighters arrived at the burning building within 5 min after receiving the alarm. In rainy weather (Case 25), or if the road into IS is blocked (Case 4 and 18), the situation will get worse.
4. Fire spread: Whether the fire will spread to the neighboring buildings is mainly related to the fire separation distance and weather factors (mainly wind speed). For some burning cases, the fire separation distance was not considered during design (Case 8, 13 and 18). Some buildings designed the fire separation, but it was occupied by combustible sundries or illegally modified, increasing the possibility of fire spread on the contrary (Case 3, 19 and 23). The wind speed was not found to aggravate the spread in these cases, but it cannot be ignored, especially for those areas with higher wind speed. Some studies have also analyzed the effects of fire separation distance and ventilation in IS [2,36].
5. Safety evacuation: From the perspective of safe evacuation, we try to fully consider the factors that affect the evacuation (See Appendix B). In these cases, the factors are in poor condition for IS. There was only one staircase and one exit, and the evacuation channel was filled with combustible materials in many houses (Case 4, 11, 16, 17, 19 and 25). In some industrial buildings, the emergency lighting system and evacuation indicator system was damaged and did not function (Case 9 and 10). Meanwhile, there are many short-term rental houses in IS, and the occupants have low awareness of fire safety and are also unfamiliar with the escape routes of the building. Once a fire occurs, it is easy to cause casualties.

## 5. Discussion

According to the above analysis, we have a semi-quantitative and staged understanding of the fire risk in IS in China. In this section, we will have a discussion of the high fire risk in IS based on the fire risk sources, including fire protection design, fire equipment and fire safety management, and then put forward some suggestions to reduce the fire risk.

### 5.1. Fire Risk Sources

- Fire protection design

**Figure 7:**
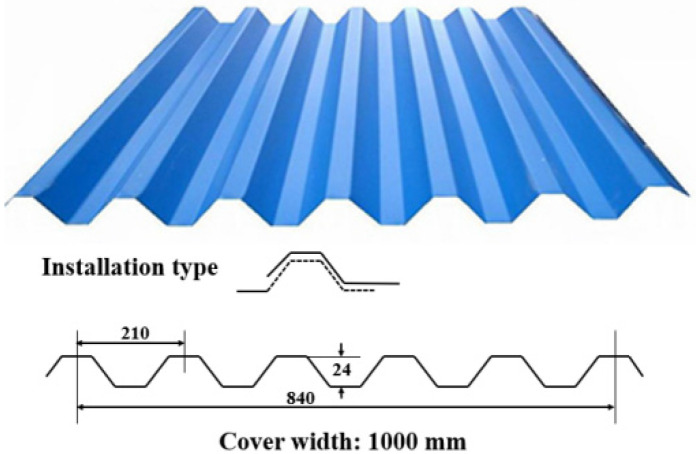
A kind of common color steel plate on the market. It is dangerous when burning.

First, in terms of building materials, many buildings in old communities use brick, adobe or wood for their walls, but these materials have low fire resistance ratings. In some buildings, the ceilings, floors and walls are wood, which will aggravate the development and spread of fire (e.g., Case 4, 8, 18 and 26). For some buildings in the informally constructed or modified settlements, the roof is made of color steel plates. Figure 7 shows a kind of common color steel plate on the market in China [37], which is cheap, convenient and beautiful for construction. However, it is a great fire hazard as it has poor fire resistance. The strength of this material will rapidly decrease and collapse easily when the building catches fire, forming a large area of combustion and producing dense smoke and high temperature [38,39,40].

**Figure 8:**
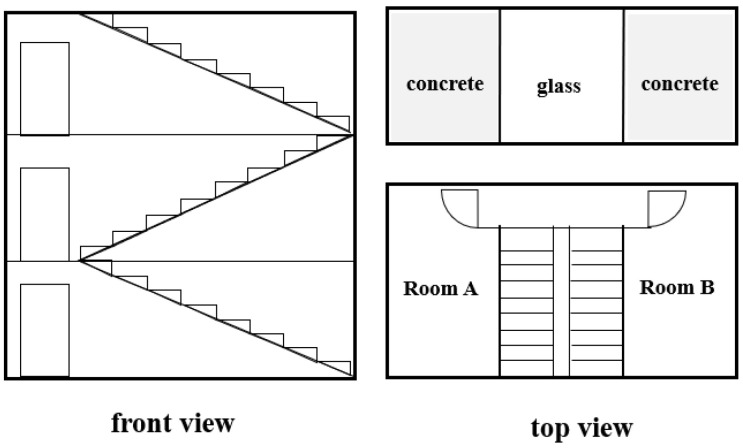
The internal structure of “Tongtian house”. In this structure, the only path to go up and down is the staircase in the middle that leads to the top.

Second, the design of fire separation and evacuation in IS is inadequate. There is a lack of fire compartment in some buildings. For example, the facade of the building in Case 2 is barbed wire instead of solid wall, and in Case 11, the partition wall between the kitchen and the hall has not reached the ceiling, which failed to prevent the fire. In many houses, there is only one staircase and one exit; even worse, the evacuation channel is filled with combustible materials such as cartons and plastic bottles or parked with bicycles/electric bicycles. When a fire occurs, they will burn and generate a lot of heat and smoke to increase the fire, but also block the evacuation route. For many burning cases, the house was built with a middle staircase and surrounded by rooms according to residents’ living habits, and the internal structure is shown in Figure 8. In this structure, the only path to go up and down is the staircase in the middle that leads to the top, and the top is closed with glass for lighting. This type of house was called “Tongtian house”, which means the house can be directly connected to the heaven in Chinese. However, once a fire occurs, the middle are with the staircase of the building will serve as a chimney. Smoke spreads rapidly from bottom to top, and once the smoke starts to enter the stairwell, the stairwell becomes the smoke spreading channel, which overlaps with the personnel escape channel, leading to the failure of evacuation. In addition, as the roof is enclosed with glass, there is a large amount of heat and toxic smoke generated in the stairwell without exhausting, making it impossible for people to survive [41].

Third, the electrical fire protection design of some buildings in IS is not standardized (e.g., Case 9, 11, 22 and 25). The laying of electrical wires or cables is hazardous as they are bare, and there is a lack of short-circuit protective devices (SCPD), or the SCPD was installed incorrectly. When sparks are generated by an electrical fault, they can easily turn into big fires if the combustibles are not separated, such as combustibles directly covered on the electrical wires, or the distance from the electrical circuit is too small.

- Fire equipment

For ordinary residential buildings, considering the affordability of different classes, the configuration of fire equipment is not mandatory in the fire regulations in China, so the actual situation is that there is no fire equipment in most residential buildings, especially in IS with low economic levels. The fire detector, for instance, is not installed in houses, which makes it difficult for people to detect fires early and take actions to extinguish them or escape, especially during the night. The reason for serious casualties in some cases is that the fire occurred at night or early morning when people were asleep, or the discovery and alarm of the fire were late, inducing the death of carbon monoxide inhalation. The fire extinguishers are also extremely lacking, and the effectiveness of extinguishing a fire by personnel is extremely low. Due to the lower floors of general residences in IS, there is no mandatory requirement of an automatic sprinkler system, so the firefighting mainly depends on the fire brigade when the fire becomes larger. However, many IS are far away from the city and the fire brigades; the fire brigade has to spend a longer time on the trip, weakening the fire control ability.

For industrial and commercial buildings, such as plants, hotels, restaurants, etc., however, the fire hydrant system and automatic sprinkler system are mandatory. In some buildings in IS, they are substandard. For example, the sprinkler system in case 1 was closed, the indoor fire hydrant was not connected to the municipal pipe network and there was no fire water tank in Case 9, and in Case 20, a component of the sprinkler system failed, causing the system to fail to function.

It should be pointed out that many burning cases in IS were originally designed as residential buildings, but they were actually turned into “mixed-function” buildings, which mixed the functions of accommodation, production, storage and business (IFS, e.g., Case 3, 5, 7, 8 and 15). For this type of building, it is not a standardized and legal single-function building, so fire equipment should be necessary. In these burning cases, there was no related fire equipment.

Moreover, some IS are located in urban–rural fringe areas or villages in the city, where the number and layout of municipal fire hydrants are improper. It will also reduce the fighting ability of the fire brigade.

- Fire safety management

Relatively, most of the residents in IS are lower-income and less-educated, and their safety awareness is low. Some studies have revealed the high fire risk related to low income and education [42,43,44]. The fire control publicity and training provided by the communities are inadequate: on one hand, people have little knowledge of requirements on safe ignition, so the use of electrical appliances or oil was improper, or the open flame was close to combustible materials. On the other hand, there is no training and drill for extinguishment or evacuation, resulting in the improper response to the fire at an early stage and incorrect escape ways when the fire becomes big; some even jumped off the building (Case 4). Similarly, the burning of industrial or commercial buildings in IS have caused serious casualties due to the insufficient emergency quality and capabilities of staff, as they were not trained in accordance with fire regulations.

In the informally functioned settlements, the mixed functions have also led to a large increase in flammable and combustible substances. In terms of management, the fire responsibility has not been cleared, and the strict fire safety operating procedures, detailed extinguishment and evacuation plans have not been formulated.

### 5.2. Solutions

In this section, we attempt to provide some suggestions to reduce the risk, respectively, for the building occupants, community organizations and emergency managers:

For the occupants, it is necessary to check the electrical system regularly and dispose the combustibles properly. Worn and aging wires need to be replaced in time, and the bared wires can be protected by flame-retardant pipe. At the same time, the combustibles should be away from electrical circuits or electrical equipment as far as possible. Some simple but useful fire equipment, such as fire detectors, fire extinguishers, gas masks and escape ropes, should also be gradually promoted to be configured within families. Since the income level of occupants in IS is relatively low, the cost of the renovation and configuration can be partly subsidized by the government to increase the coverage rate.

For community organizations, managers and volunteers can provide the basic knowledge of fire prevention and response, especially the use of fire extinguishers and safe evacuation, through regular community promotion activities, such as evacuation drills and practical fire extinguisher training. Meanwhile, through community inspections, a reminder of the occupied evacuation channel can be made, and it can be ordered to be corrected.

For the emergency managers, voluntary firefighters should be trained, and micro fire stations should be established. IS are generally far away from the city center or in rural areas, and the reliability of the professional fire brigade is reduced because of the time of the trip. It is helpful to set up micro fire stations near IS with fire nozzles, hoses, water pumps and fire extinguishers and train volunteer firefighters regularly. With these measures, firefighting and evacuation will be advanced and standardized. However, it is more important for the government to prevent the appearance of new IS by strictly reviewing the construction and gradually promote the demolition of IS in a reasonable and orderly manner if possible.

**Figure 9:**
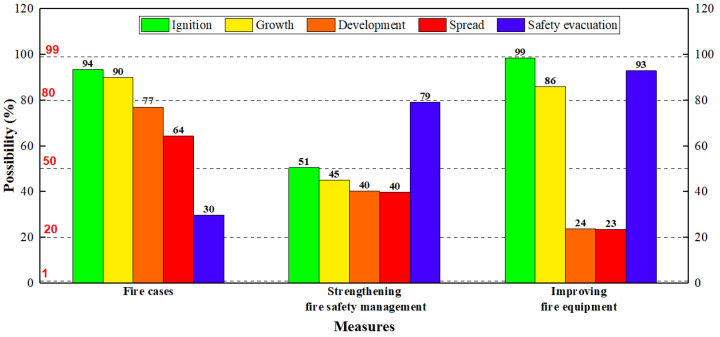
The risk change with two types of measures: strengthening fire safety management or improving fire equipment.

These suggestions can be categorized into two types of measures: strengthening fire safety management or improving fire equipment (for IS, the fire protection design can be hardly changed as the buildings have been in use, so the measures from fire protection design are not considered), and we use Bayesian networks to illustrate the effectiveness of these measures. The states of nodes remain unchanged, except for those nodes relating to fire safety management or fire equipment, and the uncertain risk will be changed accordingly. The results are shown in Figure 9. In this figure, the values of “Fire cases” are the average values of the possibilities of the 26 cases, and the values of “Strengthening fire safety management” and “Improving fire equipment”, respectively, correspond to the possibile results when only the sates of nodes relating to fire safety management or fire equipment are changed.

From Figure 9, it can be seen that strengthening fire safety management will greatly reduce the probabilities of ignition and growth, but it cannot significantly prevent the development and spread of fire; while improving fire equipment can significantly prevent the development and spread, although it cannot prevent the occurrence of fire (as unsafe behavior and combustibles still exist). Whether it is to strengthen fire management or increase fire protection facilities, the possibility of safety evacuation can be greatly increased. However, as mentioned above, it requires a certain cost to improve fire equipment, especially for fire hydrant systems and sprinkler systems. The detailed cost-benefit analysis can be carried out further, but generally, strengthening fire management is a more feasible solution for IS, as it improves building safety greatly with low investment costs relatively. Of course, if it is affordable, the configuration of fire equipment will also increase safety. At least compared to other equipment, the cost of fire detectors and extinguishers is lower, and they can be considered in IS in the future.

## 6. Conclusions

The painful fire cases in IS have brought profound lessons to fire researchers and managers in China, and the government of some countries where IS still exist should also learn from them. With the risk index and Bayesian network methods, the specific fire risk of 26 burning buildings in IS in China were assessed in this paper, and the results also revealed the high risk of the buildings in IS. First, the risk index system is used to assess the degree of fire risk of buildings in IS semi-quantitatively from the aspects of fire protection design, fire equipment and fire safety management. Then, a Bayesian network of building fire risk is established to reflect the staged risk change from ignition to spread as well as the safety evacuation. Finally, we also put forward some suggestions for occupants in IS, community organizations and emergency managers to reduce the fire risk from the aspects of fire safety management and fire equipment, which are proved to be effective with the Bayesian network method.

In this research, we conduct a risk assessment of IS in China and propose some feasible measures to reduce fire risks, aiming to provide reference for the fire management of IS and reduce the occurrence of fires and casualties. Due to current data acquisition, we only studied the burning buildings in IS, and have not yet conducted risk assessment on ordinary buildings in IS that do not catch fire. We will conduct follow-up studies on these ordinary buildings to see if there are new risk characteristics. The risk assessment method can also be improved further: the current risk index system is for all types of buildings, and a risk index system that is more applicable to IS can be constructed, considering the special characteristics of IS; in the Bayesian network, the conditional probabilities are set based on the knowledge and experience of experts, and subsequent research can be improved on the objectivity of the probabilities.

## Figures and Tables

**Table 1:** The brief information of the fire cases in IS.

Case Number	Date	City	Cause	IS Type	Casualty
1	13 January 2011	Changsha	Electric heater failure	IMS	14
2	17 January 2011	Wuhan	Unclear	OC	14
3	25 April 2011	Beijing	Electrical circuit failure	ICS	42
4	8 August 2013	Rui’an	Electrical circuit failure	OC	7
5	19 November 2013	Beijing	Electric heater	IFS	16
6	1 January 2013	Hangzhou	Arson	IMS	5
7	11 December 2013	Shenzhen	Electrical circuit failure	IFS	21
8	11 January 2014	Shangri-La	Electric heater	OC	0
9	14 January 2014	Taizhou	Electrical circuit failure	IMS	21
10	16 November 2014	Shouguang	Electrical circuit failure	ICS	31
11	29 November 2014	Cixi	Electrical circuit failure	IFS	6
12	26 December 2014	Fuyang	Unclear	ICS	11
13	21 May 2015	Xi’an	Electrical equipment failure	ICS	3
14	25 May 2015	Pingdingshan	Electrical circuit failure	ICS	45
15	2 January 2015	Harbin	Electric heater	OC	19
16	25 June 2015	Zhengzhou	Electrical circuit failure	ICS	17
17	31 December 2015	Shenyang	Unclear	OC	6
18	15 February 2016	Qionglai	Careless use of fire	OC	5
19	18 June 2016	Shanghai	Electrical circuit failure	IMS	5
20	23 September 2016	Chengdu	Electrical circuit failure	IFS	0
21	26 May 2016	Hangzhou	Careless use of fire	IMS	7
22	25 September 2017	Yuhuan	Electrical circuit failure	ICS	13
23	4 November 2017	Dunhuang	Careless use of fire	ICS	0
24	18 November 2017	Beijing	Electrical circuit failure	IFS	27
25	30 December 2017	Ganzhou	Electrical circuit failure	ICS	5
26	28 December 2018	Sanming	Careless use of fire	OC	5

**Table 2:** The score ratio of each global factor for the fire risk index.

Global Factor	Scores
fire protection design	20
fire equipment	40
fire safety management	40

**Table 3:** The correspondence between the orientation of experts and the possibility value.

Orientation	Possibility(%)
very likely	99
likely	80
possible	50
unlikely	20
very unlikely	1

## Data Availability

Not applicable.

## Appendix A

In this research, the fire risk index system is composed of 69 factors based on the fire regulations in China and, considering the availability of information and the weight of each factor, is decided by experts with the AHP method. The factors are structured under three global factors: fire protection design, fire equipment and fire safety management; the specific description and weight of each factor under the global factor are shown in Table A1, Table A2 and Table A3. There are four grades for each factor, namely A, B, C and D from high to low. When calculating the risk index, A is converted into 100 points, B is 70 points, C is 40 points and D is 0 points as $x_{i}$ in Equation (1). The description of each grade is also specified. If the information of a factor is unknown, the default score is 60 points. The information missing factors in each case did not exceed half of the total number of indicators (35), ensuring the rationality of the results.

**Table A1:** The description and weight of each factor under the global factor of fire protection design.

Objects	Factors	Weight
Building layout ^1^	Building height	0.00435
	Fire resistance rating	0.00435
	Fire separation distance	0.00102
	Fire lane setting	0.00168
	Fire climbing site	0.00168
	Fire elevator	0.00168
	Fire lane occupation	0.00168
Fire compartment	Equipment rooms	0.01468
	Fire doors, roller shutter and firewalls	0.01957
	Pipe shaft	0.01468
Design for evacuation	Evacuation routes	0.00700
	Staircase	0.02099
	Emergency exit	0.02099
	Emergency doors	0.02099
	Shelter	0.00700
Decoration and insulation materials	Combustion performance of the wall materials	0.01077
	Combustion performance of the indoor decoration materials	0.01077
	Installation type of decoration and insulation materials	0.00359
Electrical fire protection design	Separation distance of wires	0.00345
	Quality of electrical equipment and cables	0.01036
	Fire protection for wire laying	0.01036
	Fire prevention between electrical equipment and combustibles	0.00345
Exposure condition	Relative humidity	0.00123
	Wind speed ^2^	0.00368

^1^ Referring to *Code for Fire Protection Design of Building* (GB 50016-2014) for the requirement and classification of building height, fire resistance rating, fire separation distance and so on. ^2^ Referring to *Wind Scale* (GB/T 28591-2012) for detailed description of wind scale.

**Table A2:** The description and weight of each factor under the global factor of fire equipment.

Objects	Factors	Weight
Fire water supply system	Fire pump	0.02500
	Stabilized pressure pump	0.02500
	Water level in the fire water tank	0.02500
Fire hydrant system	Fire hydrant components	0.01071
	Fire hydrant start pump	0.03214
	Fire hydrant pressure	0.03214
Automatic sprinkler system	Sprinkler components	0.02250
	Model and layout of nozzle	0.00750
	End water-test equipment installation	0.02250
	End water-test equipment pressure	0.02250
Fire alarm system	Fire control center	0.00804
	Model and layout of fire detectors	0.00804
	Fire detector’s function	0.00804
	Fire protection telephone	0.00804
	Emergency broadcast	0.00804
	Graphic display devices	0.00804
	Linkage control of fire signal	0.00804
	Linkage control of fire roller shutter	0.00261
	Failure rate of fire detectors	0.00804
	Electrical fire monitoring device	0.00804
Smoke management system	Exhausting system function	0.00938
	Model and layout of exhausting system	0.00313
	Smoke prevention system function	0.00938
	Model and layout of smoke prevention system	0.00313
Emergency power supply system	Fire emergency power	0.01250
	Terminal switching device	0.01250
Emergency lighting system and evacuation indicator system	System components	0.00938
	Model and layout of luminaires	0.00313
	Model and layout of evacuation indicators	0.00313
	Linkage control function	0.00938
Fire extinguishers	Model of extinguishers	0.01500
	Layout of extinguishers	0.00500
	Validity period and pressure of extinguishers	0.00500

**Table A3:** The description and weight of each factor under the global factor of fire safety management.

Objects	Factors	Weight
Fire legality	Fire legality of the building	0.02071
Fire safety management system	Security policies and operating regulations	0.01280
	Fire protection archives	0.02231
	Responsibilities of the departments and personnel	0.02545
Implementation of fire safety management	Fire safety training and education records within one year	0.01771
	Fire inspections in the past 30 days	0.05312
	On-duty status of the control room in the past 30 days	0.05312
	On-duty status of the control room in the past 24 h	0.01771
	Routine maintenance in the past 30 days	0.05312
	Alarm review in the past 24 h	0.05312
	Rectification of fire hazards in the past 7 days	0.05312
	Plans and drill records within one year	0.01771

## Appendix B

There are a total of 66 nodes in the Bayesian network structure for fire risk assessment, which affect the possibilities of fire from ignition to spread and safety evacuation. Since there are so many nodes, to simplify the process of parameter setting and probability operation, most of the nodes are set to only two states. The name and states of each node related to each stage of the fire are shown in Table A4, Table A5, Table A6, Table A7 and Table A8. If the state of a node is unknown, it is treated as being in a certain state with equal probability. The information missing nodes in the Bayesian network for each case did not exceed half of the total number of nodes (33), ensuring the rationality of the results. There are many conditional probability tables because of the large number of nodes, and they are not shown in the text.

**Table A4:** The name and states of the nodes related to ignition in Bayesian network.

Number	Name	States
I1	Open flame	Yes; No
I2	Smoke	Yes; No
I3	Play with fire	Yes; No
I4	Arson	Yes; No
I5	Operation with sparks	Yes; No
I6	Human sources	Yes; No
I7	Unsafe electricity consumption behavior	Yes; No
I8	Standardization of electrical equipment and wires installation	Yes; No
I9	Separation from combustibles	Yes; No
I10	Electrical sources	Yes; No
I11	Pyrophoric chemical	Yes; No
I12	Dangerous substance	Yes; No
I13	Lightning stroke	Yes; No
I14	Environmental factor	Yes; No
I15	Ignition sources	Yes; No
I16	Security policies and operating regulations	Yes; No
I17	Fire safety training and education	Yes; No
I18	Rectification of fire hazards	Yes; No
I19	Fire prevention management	Yes; No

**Table A5:** The name and states of the nodes related to growth in Bayesian network.

Number	Name	States
S1	Ignition	Yes; No
G1	Fire detector’s settings	Yes; No
G2	Fire detector’s function	Yes; No
G3	Fire alarm system	Yes; No
G4	Configuration of extinguishers	Yes; No
G5	Operation training of extinguishers	Yes; No
G6	Responsibilities of the departments and personnel	Yes; No
G7	Manual extinguishing	Yes; No

**Table A6:** The name and states of the nodes related to development in Bayesian network.

Number	Name	States
S2	Growth	Yes; No
D1	Vertical fire compartment	Yes; No
D2	Horizontal fire compartment	Yes; No
D3	Fire compartment	Yes; No
D4	Standardization of decoration and insulation Materials	Yes; No
D5	Fire resistance	Good; Medium; Poor
D6	Structural fire protection	Yes; No
D7	Fire control center	Yes; No
D8	On-duty status	Yes; No
D9	Fire control manager	Yes; No
D10	Linkage control function	Yes; No
D11	Fire control system	Yes; No
D12	Automatic sprinkler system	Yes; No
D13	Fire hydrant system	Yes; No
D14	Distance to the fire station (Whether the arrival time is more than 5 min)	Yes; No
D15	Clear fire lane	Yes; No
D16	Fire brigade	Yes; No

**Table A7:** The name and states of the nodes related to spread in Bayesian network.

Number	Name	States
S3	Development	Yes; No
Sp1	Fire separation	Yes; No
Sp2	Wind speed (Whether the wind level is more than Level 4)	Yes; No

**Table A8:** The name and states of the nodes related to safety evacuation in Bayesian network.

Number	Name	States
S3	Development	Yes; No
E0	Safety evacuation	Yes; No
E1	Evacuation drills	Yes; No
E2	Responsibilities of the departments and personnel	Yes; No
E3	Evacuation skills	Yes; No
E4	Evacuation route	Yes; No
E5	Staircase	Yes; No
E6	Emergency exit	Yes; No
E7	Evacuating gate	Yes; No
E8	Shelter	Yes; No
E9	Evacuation facilities	Yes; No
E10	Emergency broadcast system	Yes; No
E11	Emergency announcements	Yes; No
E12	Smoke management system	Yes; No
E13	Building height	Yes; No
E14	Emergency power supply system	Yes; No
E15	Emergency lighting system	Yes; No
E16	Evacuation indicator system	Yes; No
E17	Fire scene environment	Yes; No
E18	Complexity of evacuation process	Yes; No
